# Introducing npj Antimicrobials and Resistance

**DOI:** 10.1038/s44259-023-00004-5

**Published:** 2023-05-10

**Authors:** Jessica M. A. Blair

**Affiliations:** Institute of Microbiology and Infection, College of Medical and Dental Sciences, University of Birmingham, Edgbaston, Birmingham B15 2TT UK

**Keywords:** Antimicrobial therapy, Antimicrobial resistance, Antimicrobial resistance, Antimicrobial resistance, Antimicrobial resistance, Antimicrobial resistance, Antimicrobial resistance, Antimicrobial responses

Our ability to treat infectious disease has saved millions of lives and has been one of the most important advances in the history of medicine. Antimicrobials now underpin all areas of modern medicine. These drugs enable successful surgery, cancer chemotherapy, organ transplantation and the survival of individuals with immunosuppressive conditions, as well as treating infection in otherwise healthy individuals. However, as usage of these critical medicines has increased, the selection for pathogens that are resistant to treatment has been an inevitable consequence.

Antimicrobial resistance (AMR) is an escalating global health crisis. The emergence of resistant strains of bacterial, fungal, viral and parasitic pathogens poses a serious threat to our ability to treat infection. Recent estimates suggest that 4.95 million people die every year from antimicrobial-resistant infections^1^, and worst-case scenario predictions suggest this figure will rise to 10 million by 2050^2^ if we continue on the current path – for context, this is one death every three seconds.

That is why we are excited to launch the first issue of *npj Antimicrobials and Resistance*, a new journal dedicated to exploring and understanding all aspects of antimicrobials and antimicrobial resistance and I am absolutely delighted to be its first Editor in Chief.

The challenges posed by antimicrobial resistance are complex and global. Therefore, our scope is intentionally broad. We will publish basic, applied, clinical or policy-based studies that advance our understanding in all areas of antimicrobials and antimicrobial resistance. We welcome studies from academia, as well as industry, and are open to studies examining AMR in the context of humans, animals, and plants.

While focus is often directed to antibiotic resistance in bacteria, the term antimicrobial also encompasses antifungal, antiviral and antiparasitic drugs and we are keen to publish articles from across this spectrum. Indeed, special content is already lined up on the topic of antifungal resistance so do look out for our call for papers. Many of the challenges and themes in each of these areas are similar and cross cutting and by providing a single destination to publish articles in each of these disciplines we hope to help to build a cohesive community that is not restricted by target organism and to encourage collaboration and sharing of ideas.

Importantly, the global AMR crisis will not be solved by scientists of any one discipline alone so *npj Antimicrobials and Resistance* is particularly keen to showcase interdisciplinary studies to help drive the field forward and encourage real innovation.

AMR is a truly global problem but the nature and scale of the crisis is not distributed equally as the greatest burden of both morbidity and mortality is found in lower and middle income countries. Therefore, we aim to publish studies from across the globe and will support authors from these regions to publish with us. Furthermore, our team of Associate Editors and the Editorial Board have been selected, not just for their outstanding and diverse expertise, but also to represent scientists from across the globe. We will continue our efforts to diversify our board as we expand in the coming months and years.

We believe that *npj Antimicrobials and Resistance* can be a vital resource in the global effort to combat antimicrobial resistance. We look forward to working with the research community to advance our understanding of this critical issue and make progress toward a world where antimicrobial resistance is no longer a threat to public health.
